# Current outlook of ethics in research with human subjects

**DOI:** 10.1590/S1808-86942011000200018

**Published:** 2015-10-19

**Authors:** Marystella Tomoe Takahashi, Henrique Faria Ramos, Carlos Diógenes Pinheiro-Neto, Ivan Dieb Miziara, Reynaldo Ayer de Oliveira

**Affiliations:** 1 Otorhinolaryngologist, Collaborating Physician from the Group of Voice - ENT Division - University Hospital - Medical School of the University of São Paulo; 2 Otorhinolaryngologist, Preceptor - Department of Otorhinolaryngology - University Hospital - Medical School of the University of São Paulo; 3 Otorhinolaryngologist; Graduate Student - Department of Otorhinolaryngology - University Hospital - Medical School of the University of São Paulo; 4 Associate Professor - Medical School of the University of São Paulo, Head of the Stomatology Group - Department of Otorhinolaryngology - University Hospital - Medical School of the University of São Paulo and Full Professor of Forensic Medicine and Medical Deontology - FMABC; 5 PhD in Pathology - State University of São Paulo - Júlio de Mesquita Filho, UNESP. PhD, Professor of Bioethics - Department of Forensic Medicine, Medical Ethics, Social Medicine and Occupational Medicine - Medical School of the University of São Paulo. Otorhinolaryngology Program - University Hospital - Medical School of the University of São Paulo

**Keywords:** bioethics, ethics committees, research, codes of ethics, principle-bases ethics, ethics, research

## Abstract

In the last decades, medical care has been increasingly permeated by the concept of evidencebased-medicine, in which clinical research plays a crucial role in establishing diagnostic and treatment. Following the improvements in clinical research, we have a growing concern and understanding that some ethical issues must be respected when the subjects are human beings. Research with human subjects relies on the principles of autonomy, beneficence, no maleficence and justice. Ordinance 196/96 from the National Health Board adds to the Brazilian legislation such renowned bioethical principles.

**Aim:**

Discuss the main ethical aspects involved in research with human subjects.

**Materials and Methods:**

Critical analysis of Ordinance 196/96 and related literature.

**Conclusion:**

Ordinance 196/96 rules research with human subjects; nevertheless, it requires more in-depth discussions regarding the informed consent, use of placebo, research with vulnerable populations and research in developing countries.

## INTRODUCTION

In 1971, American oncologist Van Renssealer Potter used for the first time the term bioethics^1^. Considering our scientific and technological progress, moral dilemmas and the transformations we’ve had in the social and political scenarios during the 60's and the 70's, all aimed at promoting a new dialogue between science and humanism^2^, stirring a debate about normative and applied ethics^3^ through a new discipline which would associate biological knowledge (bio) with human values (ethics)^1^.

Bioethics consolidation happened in 1979, with the book *Principles of Biomedical Ethics*, in which its guiding principles were established: autonomy, beneficence, no maleficence and justice^4^. Autonomy is associated with the right each person has to self-governing, which from the practical standpoint is translated into the patient's consent to participate in the proposed medical procedures. Beneficence and no maleficence correspond to the Hippocratic principles of doing good (*bonum facere*) and, first of all, not harming (*primun non nocere*), alluding to the need of always seeking the well-being and taking care of the interventions. Justice is the principle of equity, in which the equal must be treated equally and the unequal must be treated in an unequal fashion^3^.

These principles are still valid. The contemporary bioethics scenario is based on an ethics in which there is a balance between respect for people and the demands of investigation, between respect for individual values and the interest for the collective^5^.

## OBJECTIVE

The goal of the present traditional review paper is to discuss the main ethical aspects involved in research with human beings.

## MATERIALS AND METHODS

The authors made a critical analysis of codes of bioethics, focusing of Ordinance 196/96 from the National Board of Health and correlate literature.

## DISCUSSION

In recent decades, medical care has been increasingly permeated by this concept of evidence-based medicine, in which clinical research plays a crucial role in establishing diagnostic and treatment guidelines. With the progress of clinical research, we started having the concern and the understanding that certain ethical standards must be observed when the goal of the study involves human beings^6^.

In 1947, with the recognition of the so-called “crimes against humanity” perpetrated during the Second World War, the Nuremberg Code was created, establishing the first rules to govern research done with human beings. Among these rules, there is the need for a voluntary consent from the participant, prior studies in labs and with animals, and the analysis of risk and benefits that investigation could bring about, the individual's freedom to withdraw at any time from the study and the proven qualification of the researchers to do the study, among other issues^7^.

In 1964, during the 18th World Medical Assembly, the Nuremberg Code was revised and the Declaration of Helsinki was approved, which also established the rules for combined clinical research with professional care and clinical research without treatment goals^8^, and until current days it is recommended in the legislation of numerous countries and submitted to constant reviews. In the Belmont report of 1979, for the first time it was established the basic ethical principles of respecting people, beneficence and justice in the ethical make up of research.

Based on the creation of such documents, there was a growing internationalization of the methodological standards and legislative proliferation and standards, concerned with matching, in practice, individual's ethics with knowledge, the rights of mankind with social well-being^5^. During the 80's, a more elaborate document was created on the subject, called: “International Guidelines for Biomedical Research with Human Beings”. These regulatory standards are based on the subject of the study receiving and understanding information concerning the study, followed by his/ her consent; the researcher's obligations; the protection of vulnerable groups or those with reduced autonomy such as children, people with mental or behavioral disorders, prisoners, individuals from underdeveloped communities and pregnant women; in the constitution and responsibility of ethical review committees^9^.

In Brazil, it was only in 1988 that we had the first guidelines regulating experimentation with human beings, through Ordinance 01/88. In 1995, the National Health Board (NHB) decided to review it, with the goal of updating it and fill out the blanks generated by scientific development. The new resolution was created through an exemplary process of participatory construction, by a group made up of representatives from numerous social and professional areas, counting on the support of physicians, theologians, jurists, biologists, business people and representatives of the users. Ordinance 196/96 from the NHB adds to the Brazilian legislation the internationally accepted bioethical principles of autonomy, beneficence, no maleficence and justice, and it is based on the aforementioned documents, as well as in the proceedings from the Constitution of the Federative Republic of Brazil, from 1988 and the associated Brazilian legislation, here the Consumers’ Code, Civil and Penal Codes, the Statute of Children and Adolescents, and others. Thus, it aims at guaranteeing rights and duties concerning the scientific community, and regarding the subjects of the study and the state^10^.

Resolution 196/96 from the NHB stresses that “Every procedure, of whichever nature involving humans beings, which acceptance is not yet established in the scientific literature, will be considered research” and that “all research projects involving human beings involve risk”, either directly or indirectly. The research's subject has a right to full medical care and indemnity, should he/she suffer any type of damage (moral, physical, psychological, intellectual, social, cultural or spiritual) arising from the study, which must be immediately terminated should any risk or damage be foreseen to the health of the subject participating in the study, consequent to it, not covered in the informed consent form. By the same token, as soon as the superiority of one method is established over another method, the research must be terminated, offering all subjects the best treatment regimen. At the end of the study, the subjects of the study must receive the same benefits arising from the project, in terms of social return, access to procedures, products or agents from the study.

The bioethical principle of autonomy can be materialized in the need for obtaining a free and informed consent form, a document in which the individual expresses his/her authorization to freely participate in the research. In obtaining such approval, the individuals must be fully briefed in an accessible language, with a clear exposure of the existing alternative methods; discomforts and any possible and expected risks and benefits; assurance of confidentiality, secrecy and privacy; freedom to refuse to participate or withdraw his/her consent at any time during the study without any penalty or loss; means of compensation.

The attempt to protect vulnerable groups deserve special attention, advocating that research must be done with individuals under full autonomy, ruling out possibilities of dependence, subordination, coercion or intimidation. According to the Ordinance, vulnerability is defined as “the state of people or groups that for any reason or motive have their self-determination capacity reduced, especially in regards of the informed consent form”.

Ordinance 196/96 considers synonymous the conditions of reduced autonomy and vulnerability. According to Guimarães & Novaes^11^, the terms are different, because a reduction in autonomy, being transitional, definitive or voluntary, is associated with a person and it is not extensive to a group, because the expression of freedom is stated in the informed consent, given by each subject individually. The vulnerable individuals are people that because of their social, cultural, ethnicity, political, economic, and educational or health situations have their differences established among them and society turned into inequality, which impairs their capacity to freely express their will. The exacerbation of vulnerability causes the reduction or total loss of individual freedom, because the same factors which cause vulnerability contribute to prevent free choice^11^.

And why does vulnerability represent a concern in bioethics? One possible answer is that vulnerable individuals or groups of individuals are subject to exploitation. On the other hand, actions geared towards protecting vulnerable people may be seen as paternalistic, and therefore be challenged by the very group which it is intended to protect. Patients with chronic diseases or disabilities may have a drop in their self-esteem, making them fragile and, therefore, vulnerable, subject to making emotional decisions, instead of rational ones^12^, and under a situation of dependence from the researchers and the institution end up waiving their self-determination. The situation of individuals in our country is also concerning, because without true access to health care, often times they seek to participate in studies as a means to obtain access to medical care^8^.

One issue in the Ordinance that deserves further consideration is that it is prohibited to provide any form of compensation to the participating individuals, although it is allowed to compensate for all expenses. Nonetheless, the Ordinance is vague in relation to which expenses may be compensated. Would compensation for the inconvenience and the time spent be included in this reimbursement? Or only expenditures with transportation or food? In some cases, such as those involving economically less fortunate individuals, compensation may be used to persuade individuals to participate in the study against their best judgment (“excessive induction”)^9^. According to Charlesworth, those economically less fortunate have reduced autonomy, since “nobody is able to develop their personal freedom when they are stressed by poverty, stricken from basic education or if they live without public order “^13^.

In an effort for such rules to be complied with, it is mandatory for one to present the research project before the Ethics in Research Committee of the Institution. The different training and competence of the individual members of the Ethics Committee, including representatives from different fields of knowledge, representatives from users, illustrates the trend that well-educated decisions must be made about the experiment to be led by the entire society. Thus, there is an attempt to eliminate from the pure medical-scientific ground the judgment on the morality of biomedical investigation^5^.

Coordinating the set, there is the National Committee of Ethics in Research (CONEP), which acts as a normative body, of resource and coordination, and it is responsible for approving and creating complementary standards in theme areas such as human genetics, human reproduction, research with indian populations, research with foreign cooperation, research involving biosafety, research with new equipment and procedures which acceptance is still not widely accepted in the literature.

## CONCLUSION

Despite the implementation of Ordinance 196/96 and international regulatory standards, there still are some ethical issues, especially in regards of the informed consent form, the use of placebo, the participation of people under vulnerability and doing research in developing countries.

The informed consent is often times seen as a mere bureaucracy, of responsibility exemption, when the true intent of the document is the protection of freedom and the dignity of the subjects of the study^8^. Concerning the use of placebo, vulnerability and research being done in developing countries, such topics make up constant objects of discussion, especially due to the pressure of pharmaceutical giants and foreign researchers, owing to future updates of the Declaration of Helsinki and Ordinance 196/96 to uphold and comply with rights and to respect the individuals involved in the study.

The current Brazilian document is not statutory or code and, even not being a law, it carries legal force, being consistent enough to be flexible with responsibility. In short, ethics in research with humans does not work as a road map; it only guides the principles of autonomy, beneficence, no maleficence and justice, just like a compass^14^.
